# Contagious Diseases Caused by Acute Miasmata

**Published:** 1888-02

**Authors:** T. Dwight Stow


### HOMŒOPATHIC PHYSICIAN,

A MONTHLY JOURNAL OF MEDICAL SCIENCE.

"If our school ever gives up the strict inductive method of Hahnemann, we are lost, and deserve only to be mentioned as a caricature in the history of medicine."—CONSTANTINE HERING.

Vol. VIII. FEBRUARY, 1888. No. 2.

### CONTAGIOUS DISEASES, CAUSED BY ACUTE MIASMATA.

(Read before the Central New York Homœopathic Medical Society, held in Rochester, New York, December 15th, 1887.)

Webster defines contagion as: "The act or process of transmitting a disease from one person to another, by direct or indirect contract."

Dunglison. 2d. "That which serves as a medium or agency to transmit disease; pestilential influence."

3d. "The act or means of propagating influence or effects."

Dunglison, in his dictionary of medical lore, besides the definition given by Noah Webster, says: "Contagious diseases are produced either by a virus capable of causing them by inoculation, as in small-pox, cow-pox, hydrophobia, syphilis, etc.; or by miasmata, proceeding from a sick individual, as in plague, typhus gravior, and in measles, scarlatina, etc. Physicians are, indeed, by no means unanimous in deciding what diseases are contagious and what not."

Quain says: "The word 'contagion' is applied in pathology to the property and process by which in certain sorts of disease, the affected body or part causes a disease like its own to arise in other bodies or other parts. The Latin word *contagium* is conveniently used to denote in each such case the specific material, shown or presumed, in which the infective power ultimately resides."

### Analysis of § 73, Hahnemann's "Organon of Medicine," page 137, Hering's Translation, 1848.

This should be considered in connection with the preceding § 72, which may be regarded as a sort of preamble, and the points I wish to make for the benefit of the profession, are as follows :

1st. Hahnemann recognized two classes of diseases he called acute and chronic.

2d. He divided acute diseases into two distinct classes.

"The first class are sporadic, isolated, and attack single individuals, and *arise* from some pernicious cause to which they have been exposed, *e. g.*, excess of eating and drinking ; want of necessary aliment ; violent impressions of physical agents ; cold, heat, fatigue, etc., or mental excitement are the most frequent causes. But for the most part they depend upon the occasional aggravation of a *latent psoric affection* which returns to its former sleep and insensibility, when the acute affection is not too violent, or when it has been cured in a prompt manner. The others attack a plurality of individuals at once and develop themselves here and there (sporadically), beneath the sway of meteoric and telluric influence, of whose action but few persons are at the moment susceptible.

"Nearly approaching to these are those which attack many individuals at the same same, arising from similar causes and exhibiting symptoms that are analogous (epidemics), and usually (sometimes ?) *become contagious when they act upon close and compact masses of human beings.* These maladies or fevers are each of a *distinct nature*, and the individual cases which manifest themselves, *being all of the same origin*, they invariably place the patients everywhere, in one identical morbid state ; but which, if abandoned to themselves, terminate in a very short space of time, either by a cure or death. War, inundations, and famine frequently give rise to these diseases, but they may likewise result from *acute miasms*, which always reappear beneath the same form, for which reason they are designated by particular names, some of which attack man but once during life, such as the small-pox, measles, hooping-cough, the scarlet fever of Sydenham, mumps, etc., and others which may seize him repeatedly, such as the plague, yellow fever, Asiatic cholera, etc."

Hahnemann recognized in a foot-note to the above section, that true homœopathists do not depend upon the *names* of diseases as guides to the selection of remedies. Also, that in his day there was a combination of purple miliary fever and scarlatina that generally called for other homœopathic remedies than Aconite, for the miliary fever, or Belladonna for scarlatina. Also, I may add in passing, the variolous, erysipelatous, scarlatinous, and other so-called contagious diseases, appear more than once, *sometimes* twice or thrice in the same subject. Of this I have personal knowledge.

Now, let us examine the section referred to, and see if Hahnemann recognized any phenomena that support the theory I am about to advance relative to contagious diseases caused by acute miasmata. The subject selected for discussion to-day, December 15th, 1887, in the light of section 73 of *The Organon.*

This is my position: The words "contagion," or "contagious diseases" are unreliable, do not satisfactorily express the cause or character of a specific morbid condition, and on this account are misleading, hazardous, and dangerous, giving rise to bad legislation and its unwarranted and tyrannical application. That, on this account, we have a duty to perform as conservators of health, life, and that security allied to public health—proper sanitation.

Referring to the first paragraph of section 73, that relates to sporadic or isolated cases, we find no language that authorizes us to believe that Hahnemann considered sporadic cases the product of contagion or in any degree contagious. Nor does our own experience with sporadic diseases guide us to such conclusion. But Hahnemann does say, in enumerating the causes of sporadic diseases, "*that for the most part they depend* upon the occasional aggravation *of a latent psoric affection,*" etc. * * * Further on, in discussing epidemic manifestations, he says: "They attack many individuals at once, arising from similar causes, and exhibiting symptoms that are analogous, and usually become contagious when they act upon close and compact masses of human beings."

In using the word "contagious," Hahnemann evidently expressed the popular notion; yet strict construction of the whole section leads us to infer that his idea of all acute diseases was that *an internal latent psora,* call it dyscrasia or constitutional taint, as Herbert Spencer calls it, as you choose, is simply aroused to action by the debilitating or alterative action of bad surroundings—in a word, by *unsanitary* conditions.

Now, my experience and observation lead me to take a departure from the common notion of contagion, viz.: That neither the atmosphere, nor the unsanitary surroundings of sporadic cases or of endemics are strictly contagious, nor are the zymotic diseases, variola, rubeola, scarlatina, diphtheria, etc., contagious. They may *seem* to be. It seems to me *far more likely* that the filth, bad ventilation, poor food, the close and crowded quarters of human beings, in whose midst small-pox, diphtheria, cholera, and sometimes scarlet-fever, typhoid fever, the plague, etc., find a ready and deadly grip, by steadily changing the organic functions (chemical or otherwise), *give rise to the disease now recognized*, be it small-pox, cholera, diphtheria, or whatever it may.

Second. It is not a *contagium* that is to be feared and met by vaccination, isolation, high fences, or cordons of police, nor by prayer or fasting, *but an unsanitary condition*, requiring pure air, wholesome food and drink, rest, etc., with, perhaps, proper medication. In this connection it will be pertinent to state: *That an entire and radical change* of our unchristian, hateful *politico-social economy*, having for its end the *prevention of poverty*, hence the introduction of sweeping sanitary measures, as the most sane and sure preventive of zymotic diseases, sporadic, endemic, or epidemic, ought to be first considered and brought about.

Third. Again, when a large number of persons, having the same or similar constitutional taints or idiosyncrasies, "latent chronic miasm," as Hahnemann expresses it, are acted upon by meteoric or telluric influences, within a certain radius, an acute endemic, or epidemic, attracts attention, and often gives rise to popular anxiety and fear, and to overt acts, legal (?) or otherwise. Such epidemics bear the precise and peculiar characteristics of the constitutional peculiarities of the individuals composing the group; *i. e.*, according to the systematic taint will the crop be—the genius epidemicus exciting and the constitutional taint giving direction to the malady.

Furthermore, these *spread* not by contact but by reason of similarity of condition, tendency, and surroundings.

Fourth. Such epidemics often recur in cycles of time, in obedience to fixed laws, and are in harmony with the gross and unpardonable violations of natural law so conspicuously and alarmingly visible.

Fifth. External or "acute miasms" are those agencies or forces bred and existing external to the individual, but likely to be inhaled, imbibed, or absorbed, or may be, acting magnetically or electrically—capable of lighting up an internal chronic miasm, or of so preventing normal functions as to develop disease, such as the inhalation of noxious gases, sickening odors, etc. *A clear distinction* must be made here between these infinitesimal, imponderable, intangible forces, and those tangible *vehicles* of destructive force, dependent upon inoculation for the exhibition of their morbific qualities; *e. g.*, syphilis, sycosis, gonorrhœa, pyæmia, variola, vaccinia, the poison of serpents, rabid animals, and of various vegetable and mineral poisons. The former may be infectious, the latter are the only strictly contagious diseases, *and require absolute contact*, not with the skin only, but with the blood or with nerve fibre to show their disorganizing qualities.

Here I venture the opinion that the very worst diseases entailed upon the human race are those transmitted by inoculation, syphilis, sycosis, pyæmia, hydrophobia, etc., and that by the process of vaccination, which is a sort of inoculation, we are multiplying and intensifying human misery.

Having taken the foregoing general view of the subject in hand for exciting inquiry, and the further purpose of pointing a moral, I add the following distinct statements:

(First). The medical profession is not a unit on the question of contagion or infection: indeed, there is the widest, if not the wildest, diversity of opinion on the subject. Compare the definitions of contagion and of infection, given by Dunglison and Quain, and of other writers.

(Second). It is the year of our Lord 1887, and while important discoveries, astonishing inventions, rapid communication, etc., are characteristic of the times, there is not nearly the knowledge of the etiology of diseases, particularly of the zymotic, so-called, as certain facts and comparison of facts and conditions warrant. Since, in the management of diseases called contagious, there has come to be so much arbitrary power exercised by the State over the bodies, lives, and liberty of the people, a far more correct knowledge of the whence, the what, the why, of the "zymosis" ought to obtain. The legislature, in matters medical, but reflects the opinions of "medical experts," and if in the matter of "contagious" diseases, such experts have no more and no better knowledge of the quality or essential spirit of such so-called infection or contagion than their thesis and statements betray, how baseless and arbitrary must be such legislation! We need not go far nor expend much time in ascertaining the drift of such absurd ideas, cropping out as they do in the acts of every session of the legislature, and the tyranny exercised under cover of such acts!

Sixth. Contagion is one thing, infection quite another. I deny the possibility of knowing what "*contagion*" is, and doubt its existence! Nor is the idea of infection so clear or satisfactory *that we can intelligently or positively affirm.* That meteoric or telluric influences, singly or combined, have the power of changing or perverting animal functions, I do not doubt. Cold, heat, electric influences, want, fear, fright, bad ventilation, poor food, etc., are, doubtless, the best expressions of finite beings in their efforts to get at the proximate, tangible causes of certain diseases, but are poor measures of the *subtle forces* galvanized into life within the organism, by the former physical conditions.

Experience, though bitter and expensive, has learned us this, in regard to startling endemics and epidemics: That certain conditions and habits of life, such as squalor, crowding, want, exposure, favor and intensify them. The course to pursue, then, is to prevent, not to palliate, by teaching those laws of hygiene, upon the observance of which health and life depend. The simple observance of the Golden Rule in all social and political life will surely solve the problem. Riches on the one hand, poverty the outgrowth of the concentration of riches, a Pandora's box of evils, surely following them, on the other, are the penalties of trampling the Golden Rule under foot. Anything short of radical reformation will be futile and confusing. No amount of tinkering will do.

Reasons why I reject the present " Theory of Contagion and of Contagious Diseases:"

First. Original cases, whether sporadic or epidemic, come not by exposure of person to person, but from internal changes, and sporadic cases often manifest themselves in the midst of comfort and cleanliness. They are also innocuous.

Second. It is but *the few*, taken from the ranks of the many, that become victims of the so-called zymotic or contagious diseases. The great majority of people, even though exposed to such miasms, escape. If there existed an absolutely *contagious* principle, the majority, if not all, would be victimized. And these *are the special few*, whose constitutional peculiarities, idiosyncrasies, or within whom are " latent chronic miasms," similar to or having an affinity for the external acute, who are, by reason of such, more susceptible to morbific influences.

Third. Physicians and nurses are directly and often exposed to the so-called contagious diseases, and in going to and fro, expose themselves and others, even their own families, yet with rare exceptions, enjoy perfect immunity.

Fourth. We all know how often it is the case that malignant or grave cases of diphtheria, scarlet fever, measles, and typhoid fever attack but one or two of a large family, yet they breathe the same atmosphere, eat food from the same dish, wash, join hands, and, during the period of incubation, sleep together. Surely, if the theory of contagion be true—and judging by the arbitrary measures often taken in the course of epidemics, people deem it true—we should confidently expect no such limitation of cases.

Fifth. It is further asserted by pathologists that "germs" of disease, once lodged within the organism, are capable of multiplication and *do* multiply. This is expressive of quantity, of substance; and Dr. Letheby informs us that such germs or molecules are the one hundred thousandth part of an inch in diameter! But Baron Liebig, Beale, and others, tried microscopes, great and small, and chemical tests, but found no such evidence. I submit that disease-producing "germs or molecules" exist only in the imagination; but that life-giving, life-destroying, subtle force—the *soul* of matter—is beyond the pale of microscopes or chemical tests.

We do not know, hence *we cannot affirm* the existence of contagion, and on this account have no right to invade the sanctity of person or home on the plea of public safety, for safety lies in another direction. Sanitation, with more freedom, is the true policy and "the safe side!"

Very instructive quotations might here be offered by introducing a speech made by Sir Clark J. Jervoise, Bart., in the British Parliament, on "Infection," had we space.

Sixth. The theory of the rapid multiplication of "disease germs" once absorbed, is *extremely improbable;* for, if it were true the death-rate would be unlimited, so great and paralyzing would be the effects, recovery would be the exception, not the rule. The probability is that the disease-producing "force" exhausts itself and recovery follows, or the patient succumbs to the shock.

Seventh. Certain diseases, like Asiatic cholera, small-pox, measles, hooping-cough, have characteristic peculiarities. Cholera is essentially a summer and fall disease, disappearing when frosts come; so of yellow fever. Small-pox appears in the spring and fall; sometimes in the winter, and seeks out the places, whether in the tented field or in the close quarters of the poor of crowded cities; and *there* we find its greatest activity, often amounting to an epidemy in such places. These facts are significant; ought to open our eyes to the fact that not a contagious or infectious principle is the *prima-causa-morbi*—but certain habits of life, *e. g.,* the crowding of human beings together, the want of food, and uncleanliness, so disturb the functions of respiration, digestion, and assimilation, and excretion from the skin and kidneys, that the blood, charged with a peculiar morbid product, finds an outlet for said product through the kidneys, skin, and mucous membranes. Each and every person similarly situated will finally succumb to such surroundings—sooner or later—no others will. Isolated, sporadic cases *spring from the same internal changes*, though the environments be entirely dissimilar. The fact that in the course of an epidemic invasion, we find and designate certain districts or quarters "infected," while other localities are unaffected, is proof enough of the peculiar *limitation* of the particularly manifest disease: limited *because of environments.* The environments, including bad food, uncleanness, provoking the *internal specific changes.*

But, in the single, isolated case, whose surroundings may be all that is desirable, gluttony, intemperance, loss of sleep and irregular habits, anxiety, etc., may be the causes of the changes of function, resulting in small-pox, scarlet fever, diphtheria, etc. In either case, there has been a violation of some or many natural laws. In the one case—that of the poor—the environments are forced upon him by society; in the case of the well-to-do, it is willful or accidental.

Eighth. "Zymotic" diseases are those supposed to arise from the decay and fermentation of animal or vegetable matter; they are also considered peculiarly contagious or infectious. They are for the most part as follows:

Asiatic cholera, cholera-morbus, erysipelas, erythema, epidemic dysentery, rubeola, scarlatina, diphtheria, plague (now nearly extinct), typhoid or enteric fever, variola and its modifications, and by some nosologists, pertussis, and meningitis. But opinions differ widely as to their source and their "contagious" qualities. With the single exception of cholera, they are febrile diseases, and bear the marks of some dyscrasia or constitutional disturbance. What is this dyscrasia? What is the constitutional disturbance?

Gentlemen, I have more than a faint suspicion, *a well-founded belief*, that it is a *latent force* engrafted upon the race by *inoculation*, including vaccination—the "latent psora" of Hahnemann.

That these forces, at times latent, at times active, produce the various phenomena of the several diseases termed zymotic, contagious, infectious; that they are transmissible by heredity and inoculation. If my ideas thus arranged and communicated to you, my fellow-physicians, be reasonable, harmonious, and true, or if they approximate the truth, then let me *hope* that you will further investigate them, and as custodians of public health, give them such practical application as shall from time to time be related to the public weal, in dealing with the "zymoses" and so-called infectious diseases.

Recollect, "An ounce of prevention is better than a pound of cure." Sanitation (including homœopathic medication), *nothing else*—will wipe out so-called contagious diseases.

Respectfully submitted,

T. Dwight Stow.

